# Effect of *Thymbra capitata* (L.) Cav. on Inflammation, Senescence and Cell Migration

**DOI:** 10.3390/nu15081930

**Published:** 2023-04-17

**Authors:** Jorge M. Alves-Silva, Sónia Pedreiro, Carlos Cavaleiro, Maria Teresa Cruz, Artur Figueirinha, Lígia Salgueiro

**Affiliations:** 1 Institute for Clinical and Biomedical Research, University of Coimbra, Health Sciences Campus, Azinhaga de S. Comba, 3000-548 Coimbra, Portugal; jmasilva@student.ff.uc.pt; 2 Faculty of Pharmacy, University of Coimbra, Health Sciences Campus, Azinhaga de S. Comba, 3000-548 Coimbra, Portugal; uc2007119618@student.uc.pt (S.P.); cavaleir@ff.uc.pt (C.C.); trosete@ff.uc.pt (M.T.C.); amfigueirinha@ff.uc.pt (A.F.); 3 Associated Laboratory for Green Chemistry (LAQV) of the Network of Chemistry and Technology (REQUIMTE), University of Porto, 4099-002 Porto, Portugal; 4 Chemical Process Engineering and Forest Products Research Centre, Department of Chemical Engineering, Faculty of Sciences and Technology, University of Coimbra, 3030-790 Coimbra, Portugal; 5 Center for Neuroscience and Cell Biology, Faculty of Medicine, University of Coimbra, Rua Larga, 3004-504 Coimbra, Portugal

**Keywords:** aging, phytochemical composition, inflammatory mediators, essential oil, hydrodistillation residual water

## Abstract

Aromatic plants are reported to display pharmacological properties, including anti-aging. This work aims to disclose the anti-aging effect of the essential oil (EO) of *Thymbra capitata* (L.) Cav., an aromatic and medicinal plant widely used as a spice, as well as of the hydrodistillation residual water (HRW), a discarded by-product of EO hydrodistillation. The phytochemical characterization of EO and HRW was assessed by GC-MS and HPLC-PDA-ESI-MS^n^, respectively. The DPPH, ABTS, and FRAP assays were used to disclose the antioxidant properties. The anti-inflammatory potential was evaluated using lipopolysaccharide-stimulated macrophages by assessing NO production, iNOS, and pro-IL-1β protein levels. Cell migration was evaluated using the scratch wound assay, and the etoposide-induced senescence was used to assess the modulation of senescence. The EO is mainly characterized by carvacrol, while the HRW is predominantly characterized by rosmarinic acid. The HRW exerts a stronger antioxidant effect in the DPPH and FRAP assays, whereas the EO was the most active sample in the ABTS assay. Both extracts reduce NO, iNOS, and pro-IL-1β. The EO has no effect on cell migration and presents anti-senescence effects. In opposition, HRW reduces cell migration and induces cellular senescence. Overall, our study highlights interesting pharmacological properties for both extracts, EO being of interest as an anti-aging ingredient and HRW relevant in cancer therapy.

## 1. Introduction

Inflammation plays a fundamental role in aging and in age-related diseases due to its interaction with the remaining hallmarks of aging, such as stem cell exhaustion, cellular senescence, mitochondrial dysfunction, loss of proteostasis, altered intercellular communication, mitochondrial dysfunction, among others [1]. More recently, inflammation has been considered a new hallmark of aging [2]. This age-related inflammatory state, often denominated inflammaging, is characterized by a low-grade, chronic, and systemic inflammation, with increased levels of circulating proinflammatory mediators and a change towards cellular senescence [3]. Inflammaging is believed to contribute to the development of many age-associated conditions. Indeed, this state is associated with increased mortality and morbidity in the elderly due to the association with the development of age-related diseases, such as type 2 diabetes, obesity, and neurodegenerative and cardiovascular diseases [1,4].

Cellular senescence is relevant in several physiological processes, including wound healing, but also in the development of age-related diseases [5]. Indeed, it has been shown that senescent fibroblasts are required for optimal wound healing [6]; however, these senescence—clearance–regeneration events are often compromised in pathological conditions and in aging, therefore causing the accumulation of senescent cells in the tissues [7]. These cells exhibit a senescence-associated secretory phenotype (SASP) that contributes, for instance, a delay in skin wound healing [8]. Cellular senescence is triggered by a variety of stimuli, including mitochondrial dysfunction, causing the production of reactive oxygen species (ROS) and the activation of the NRLP3 inflammasome [4]. Furthermore, ROS production can also be associated with the cellular response to inflammation [9]. The link between ROS and inflammation with cellular senescence is thought to be mediated by ASK1/p38 MAPK and SASP/JNK pathways [10].

Indeed, it has been shown that the blockage of NRLP3 [11] or NF-κB [12] signaling pathways delays the onset of age-related diseases, thus strengthening the link between inflammation and cellular senescence. Since senescent cells shape the outcome of several physiological and pathological processes, pro-senescent and anti-senescent therapies are actively being explored. Pro-senescent therapies can be useful in cancer treatment, while anti-senescent strategies might be useful in avoiding the accumulation of senescent cells during aging [7]. In this context, aromatic and medicinal plants arise as potential sources of anti-aging compounds due to their antioxidant and anti-inflammatory properties. Accordingly, several reviews highlighted the potential of phytochemicals in the management of aging and age-related diseases [13,14]. Although several pathways can be targeted by phytochemicals [14], NF-κB seems to be at the forefront [15]. Furthermore, the beneficial effect of nutrition in the prevention of inflammation and inflammaging has been recently highlighted [16], with a particular interest in the Mediterranean diet, associated with the intake of aromatic and medicinal plants. Indeed, adhesion to this type of diet has been associated with a lower likelihood of frailty [17]. Among all the classes of phytochemicals with relevant properties in this field, phenolic compounds and terpenoids stand out as potential anti-aging compounds [13,14]. *Thymbra capitata* (L.) Cav. (syn. *Thymus capitatus* (L.) Hoffmanns; *Coridothymus capitatus* (L.) Reichenb. (F.)) stands out due to its richness in non-volatile phenolic compounds, such as rosmarinic acid [18], and volatile molecules, such as carvacrol [19]. *T. capitata* (Lamiaceae) is a perennial aromatic plant widely distributed in the Mediterranean region [20,21,22]. The aerial parts of this plant are widely used for culinary and ornamental purposes [19] but also in the medicinal field, particularly in deregulated inflammatory conditions, skin complications, and wound healing [20,21,23,24]. Furthermore, *T. capitata* essential oil (EO) collected in Portugal has demonstrated relevant antimicrobial activity [25,26], thus highlighting the industrial value of this species. However, phytochemical studies performed with aqueous extracts of *T. capitata* collected in Portugal are scarce. In order to fill this gap, the aim of this work is the characterization of the essential oil as well as the hydrodistillation residual water (HRW), a usually discarded by-product of essential oil hydrodistillation, by GC-MS and HPLC-PDA-ESI-MS^n^. Furthermore, having in mind the traditional uses ascribed to this plant, anti-inflammatory and wound-healing activities will be disclosed. In addition, to further promote interest in this species, antioxidant effects and modulation of cell senescence will also be highlighted, thus enhancing the sustainable industrial exploitation of this plant.

## 2. Materials and Methods

### 2.1. Plant Material and Hydrodistillation

*Thymbra capitata* (L.) Cav. plant was collected in May 2021 at Carvoeiro, Algarve, Portugal, and a voucher specimen (LS 196) was deposited in the herbarium of the Faculty of Pharmacy—University of Coimbra. Plants were air-dried in the dark prior to use.

The EO and HRW were prepared by submitting the air-dried material to hydrodistillation for 3 h, using a Clevenger apparatus, in accordance with the European Pharmacopoeia [27]. After 3 h, the EO and HRW were collected. Posteriorly, the HRW was filtered under vacuum, concentrated in a rotavapor at 40 °C, frozen, freeze-dried, and kept at −20 °C in the dark until use.

### 2.2. Chemical Characterization of the EO and HRW

#### 2.2.1. GC-MS

The essential oil was analyzed by gas chromatography (GC) and gas chromatography coupled to mass spectrometry (GC/MS), as previously described by our group [28].

The identity of the volatile compounds was achieved by their retention indices (RI) on two GC columns (SPB-1 and SupelcoWax-10) and mass spectra. RI were matched with those from a home-made database and/or from literature data [29,30,31,32]. Mass spectra were compared with reference spectra from our own database, Wiley/NIST library [33], and literature data [29,31]. Relative amounts were determined based on raw data areas without a response factor correction for flame ionization detection.

#### 2.2.2. HPLC-PDA-ESI-MS^n^

The chemical characterization of HRW was performed in a high-performance liquid chromatograph (HPLC) (Finnigan Surveyor, THERMO, Waltham, MA, USA) with a photodiode array detector (PDA) (Finnigan Surveyor, THERMO) and a linear ion trap mass detector (LIT-MS) (LTQ XL, Thermo Scientific, Waltham, MA, USA). The reverse phase chromatographic column (Waters Spherisorb ODS2, Waters Corp., Milford, MA, USA) with 150 × 2.1 mm and 3 μm particle size. Solvent A (2% (*v*/*v*) aqueous formic acid) and solvent B (acetonitrile) were used as mobile phases with a gradient of 0–60 min, 5–50% (*v*/*v*) B. The flow rate was 200 μL/min at 20 °C. The detection was made in the diode array spectrophotometer using 280 and 320 nm as preferred wavelengths. The second detection was made by the mass spectrometer in negative electrospray ionization (ESI) mode generating a full mass (MS), MS2, and MS3 spectrum of the most abundant ion. Helium was used as the collision gas, with a collision energy of 35%. Nitrogen was used as nebulizing gas, with a sheath gas flow of 35 (arbitrary units), and as auxiliary gas with a flow of 20 (arbitrary units). The capillary temperature was 275 °C. Capillary and source voltage was −35.00 V and 5.00 kV, respectively.

### 2.3. Antioxidant Activity

#### 2.3.1. 2,2-Diphenyl-1-picrylhydrazyl Radical Scavenging Assay (DPPH)

The antioxidant activity by radical-scavenging activity of EO and HRW of *T. capitata* was assessed by DPPH radical scavenging assay [34]. The radical scavenging activity of the samples (10 µL) was evaluated in a reaction media containing 140 µL of methanol, 50 µL of DPPH 500 µM in methanol, and 100 µL of acetate buffer 100 mM at pH 6.0. The mixture (300 µL) was kept at room temperature, in the dark, for 30 min. The absorbance was then measured at 517 nm in a microplate reader photometer (Thermo Scientific Multiskan FC, Waltham, MA, USA). The tested concentrations range for EO were 33 to 500 µg/mL and HWR 10 to 70 μg/mL. All the determinations were performed in three independent assays in duplicate. The % of reduction of DPPH was calculated using Equation (1):

(1) $$\mathrm{Reduction}\;\mathrm{of}\;\mathrm{DPPH}\;(\%)\;=\;100-\frac{\mathrm{Abs}\;\mathrm{sample}\;-\;\mathrm{Abs}\;\mathrm{control}}{\mathrm{Abs}\;\mathrm{control}}$$

IC_50_ values were calculated from the graph % of DPPH reduction vs. logarithm of the concentration using the GraphPad Prism program (version 5.02, GraphPad Software, San Diego, CA, USA). Trolox was used as a positive control, and the results were also expressed as TE (Trolox equivalents).

#### 2.3.2. 2,20-Azinobis-(3-ethylbenzothiazoline-6-sulfonate) Assay (ABTS)

The ABTS assay was performed according to the methods used by Re et al. [35]. An aqueous solution of 7 mM ABTS^●+^ and 2.45 mM potassium persulphate (Merck, Darmstadt, Germany) was prepared. After 16 h, in the dark at room temperature, this solution was diluted with phosphate-buffered saline (PBS) at pH 7 to achieve an absorbance of 0.7 ± 0.02 at 734 nm. The ABTS assay was performed by adding 50 µL of the extract to 2 mL of the ABTS ^●+^ solution and vortexed for 10 s. After an incubation of 4 min, the absorbance of the reactional mixture was measured at the wavelength of 734 nm. The IC_50_ value was calculated from the interpolation of the graph % of ABTS vs. concentration in µg/mL. Trolox was used as a positive control, and the results were also expressed as TE (Trolox equivalents). Three independent experiments in duplicate were performed for each sample.

#### 2.3.3. Ferric Reducing Antioxidant Power Assay (FRAP)

The FRAP assay was performed according to the methods used by Benzie et al. [36]. The FRAP reagent was prepared using 300 mM acetate buffer, 10 mM of TPTZ solution in HCl 40 mM, and 20 mM FeCl_3_.6H_2_O in a proportion of 10:1:1 (*v*/*v*/*v*). The assay was performed by mixing 3 mL of the FRAP reagent with 100 µL of extract. After 6 min in the dark at room temperature, the absorbance was measured at 593 nm. Trolox was used as a positive control. The results were expressed as TE (Trolox equivalents). For each sample, three independent experiments in duplicate were performed.

### 2.4. Cell Culture

The cell lines RAW 264.7 (mouse leukemic macrophage cell line) and NIH 3T3 (mouse embryonic fibroblast) were obtained from the American Type Culture Collection (ATCC TIB-71 and ATCC CRL-1658, respectively) and were cultured as previously described by the team [37].

### 2.5. Cell Viability

The effect of different concentrations of the EO and HRW on macrophage and fibroblast viability was evaluated through the resazurin reduction test, as previously reported [38].

### 2.6. Anti-Inflammatory Potential

#### 2.6.1. Nitric Oxide Production

The capacity of the EO and HRW to decrease the nitric oxide production in lipopolysaccharide (LPS)-stimulated macrophages was assessed using the methodology described in our group [28].

#### 2.6.2. Western Blot Analysis of Pro-Inflammatory Mediators

RAW 264.7 cells (1.2 × 10^6^ cells/well) were cultured in 6-well plates and stabilized overnight. Cells were then subjected to 1 h incubation with EO (128 µg/mL) or HRW (400 µg/mL), followed by 24 h of LPS activation (50 ng/mL). Negative and positive controls comprising untreated or LPS-treated cells, respectively, were included. Cell lysate preparation followed the protocol previously performed by Zuzarte et al. [39].

The protein levels of the inducible nitric oxide synthase (iNOS) and IL-1β pro form (pro-IL-1β) were assessed by Western blot, as previously described [40]. For protein separation, an electrophoretic run with 10% (*v*/*v*) SDS-polyacrylamide gels at 130 V was performed for 1.5 h. Protein lines were consequently blotted, during 3 h at 400 mA, to membranes of polyvinylidene fluoride, which were previously activated with methanol. The membranes were then incubated for 1 h at room temperature with 5% (*w*/*v*) skim milk in TBS-T. They were further incubated overnight at 4 °C with specific anti-iNOS (1:500; MAB9502; R and D Systems, Minneapolis, MN USA) or anti-pro-IL-1β (1:1000; ab9722; Abcam, Cambridge, UK) antibodies. Finally, they were washed for 30 min with TBS-T (10 min, 3 times) and incubated for 1 h at room temperature with secondary antibodies (1:40,000; Santa Cruz Biotechnology, Dallas, TX, USA) conjugated with horseradish peroxidase. The immunocomplexes detection was performed by a chemiluminescence scanner (Image Quant LAS 500, GE, Boston, MA, USA). Antibody against tubulin (1:20,000; Sigma, Burlington, MA, USA) was used as a loading control. ImageLab software version 6.1.0 (Bio-Rad Laboratories Inc., Hercules, CA, USA) was used for protein quantification.

### 2.7. Cell Migration

The effect of the samples on cell migration was investigated through the scratch wound assay according to the methods used by Martinotti et al. [41] with slight modifications, as previously reported [40], and the open area was quantified using an ImageJ/FIJI plugin [42].

### 2.8. Etoposide-Induced Senescence

Senescence was evaluated using the senescence inducer etoposide, as reported elsewhere [43], with some modifications. Briefly, after 24 h of fibroblast culture in the presence of etoposide, cells were further incubated for 72 h, in the presence or absence of the EO (128 µg/mL) and HRW (400 µg/mL). Beta-galactosidase was assessed using a commercially available kit according to the manufacturer’s protocol (#9860, Cell Signalling Technology Inc., Danvers, MA, USA). The distinct blue color staining indicates beta-galactosidase activity. After color developments, wells were photographed for subsequent image analysis. ImageJ software version 1.53t was used for the quantitative analysis by assessing the percentage of senescent cells.

### 2.9. Statistical Analysis

The experiments were performed at least in duplicate for three independent experiments. Mean values ± SEM (standard error of the mean) are presented in the results. Statistical significance for cell viability and for the senescence and inflammatory assays was evaluated by one-way analysis of variance (ANOVA) and Dunnett’s post hoc test using GraphPad Prism version 9.3.0 (GraphPad Software, San Diego, CA, USA). While the statistical significance for cell migration assays was assessed by two-way ANOVA followed by Sydák’s multiple comparison test. *p* Values <0.05 were accepted as statistically significant.

## 3. Results

### 3.1. Phytochemical Characterization of the EO

The hydrodistillation process produced essential oil with a yield of 1.8% (*v*/*w*). Chemical characterization of the essential oil by GC and GC-MS identified 97.8 of all the compounds in the EO, as shown in Table 1. Carvacrol (79.5%) was the major compound of the EO, followed by *p*-cymene (4.8%) and γ-terpinene (4%), accounting for 88.4% of the whole essential oil.

**Figure 1:**
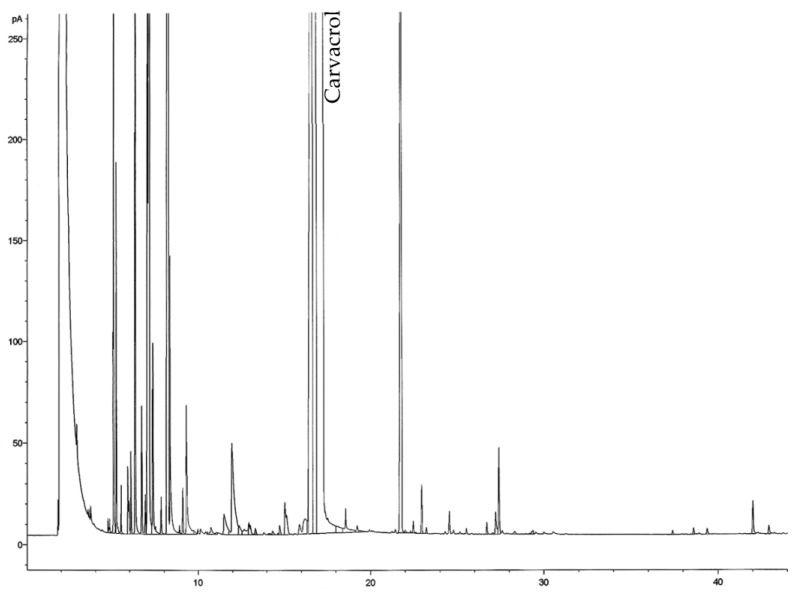
GC-FID chromatogram of EO of *Thymbra capitata* on the SPB-1 column.

GC-FID chromatogram of EO on the SPB-1 column is shown in Figure 1. Carvacrol, the predominant compound of the oil, is marked on the chromatogram.

### 3.2. Phytochemical Characterization of HRW by HPLC-PDA-ESI-MSn

**Figure 2:**
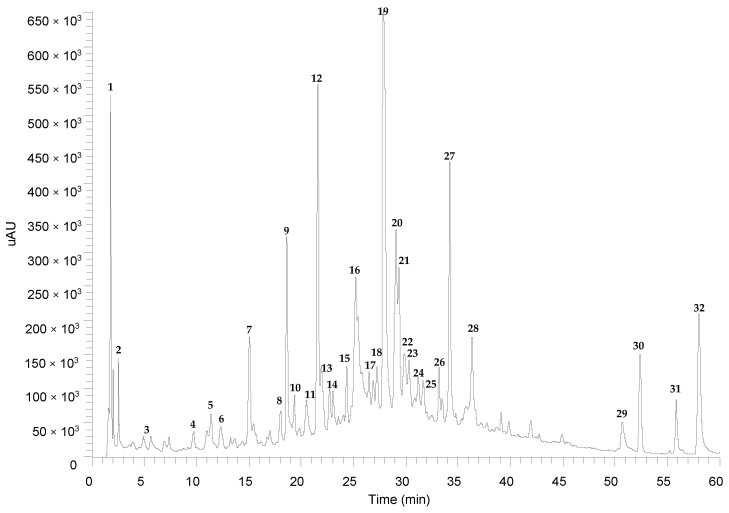
HPLC-PDA-ESI-MSn chromatogram of hydrodistillation residual water from *T. capitata*, recorded at 320 nm.

*T. capitata* hydrodistillation residual water (HRW) was characterized relative to its phytochemical composition by HPLC-PDA-ESI-MSn (Figure 2 and Table 2).

The HRW is composed mainly of phenolic acids, rosmarinic acid being the main phytoconstituent, followed by salvianolic acids J, B, E, and A. Although in lower quantity relative to phenolic acids, HRW also has flavonoids, namely flavones, flavonols, flavanones, and flavanonol derivatives. Peaks **25**, **31**, and **32** were not identified.

#### 3.2.1. Organic and Phenolic Acids

Peaks **1** and **2** presented a molecular ion [M − H]^−^ at *m*/*z* 191, yielding an MS2 base peak fragment of *m*/*z* 111 (also observed in MS3) as a result of dehydration and decarboxylation ([M − H − 2H_2_O − CO_2_]^−^) [13], and a fragment of *m*/*z* 173 that correspond to dehydration ([M − H − H_2_O]^−^). According to the literature, this fragmentation pattern is easily misunderstood between quinic acid and citric acid [44,45]. However, the relative abundance of fragments at *m*/*z* 111 and 173 can be used to distinguish these two compounds. Herein, we tentatively identified peaks **1** and **2** as citric acid isomers due to the presence of the base peak at *m*/*z* 111 [13] and its relatively higher ratio to fragment *m*/*z* 173 [45].

Peak **3** presents an absorption maximum of 280 nm and a molecular ion [M − H]^−^ at *m*/*z* 197, yielding the MS2 and MS3 base peaks at *m*/*z* 179 and 135 due to dehydration ([M − H − H_2_O]^−^) and decarboxylation ([M − H − H_2_O − CO_2_]^−^), respectively. According to these spectral features, extensively described in te literature, peak **3** was tentatively assigned as danshensu [46].

Peaks **4** and **5** share the same UV spectra profile, characteristic of hydroxycinnamic acids exhibiting two bands in the range of 285–330 nm [34]. Relatively to their mass fragmentation pattern, both displayed a molecular ion [M − H]^−^ at *m*/*z* 313 and MS2 base peak of *m*/*z* 269 (also observed in MS3) due to decarboxylation of molecular ion ([M − H − CO_2_]^−^). Additional fragment *m*/*z* at 203 is also observed ([M − H − 110]^−^) due to the loss of a catechol unit. Based on similar behavior found in previous literature, these peaks were tentatively identified as salvianolic acids F isomers [47,48].

Peak **6** was tentatively assigned as vanillic acid due to its molecular ion [M − H]^−^ at 167 and MS3 fragment at *m*/*z* 123, resulting from the decarboxylation ([M − H − CO_2_]^−^), which is consistent with its MW of 168 and fragmentation pattern previously described for this compound [49].

Peak **7** showed a UV spectrum profile characteristic of caffeic or ferulic acid derivatives, with an absorption maximum of 322 nm and a shoulder at 291 nm [34]. The molecular ion [M − H]^−^ occurred at *m*/*z* 179 and yielded an MS2 and MS3 peak base at *m*/*z* 135, indicating the loss of a carboxyl group ([M − H − CO_2_]^−^). This spectral behavior suggests the presence of caffeic acid (MW 180) [50].

Peaks **8** ([M − H]^−^ at *m*/*z* 357) and **11** ([M − H]^−^ at *m*/*z* 555) differ by 198 mass units, probably a danshensu unit. The pseudomolecular ion of Peak **8** loses one carboxyl unit yielding an MS2 base peak at *m*/*z* 313. This fragment generates fragments at *m*/*z* 295, 269, and 203, corresponding to the loss of water, carboxyl, and catechol units, respectively. This spectral behavior is characteristic of prolithospermic acid [51]. In peak **11**, the MS2 base peak at *m*/*z* 339 results from the dehydration of pseudomolecular ion ([M − H − H_2_O]^−^). The base peak in the MS3 spectrum exhibited a fragment at *m*/*z* 295 due to the loss of a carboxyl unit. The fragmentation pattern of peak **11** is suggestive of salvianolic acid K, in accordance with Wojciechowska et al. (2020) [55].

Peaks **12** and **26** have the same pseudomolecular ion [M − H]^−^ at *m*/*z* 537, corresponding to a molar weight (MW 538). In peak **12,** an MS2 base peak at *m*/*z* 339 occur, indicating the loss of a danshensu unit ([M − H − 198]^−^), and an MS3 base peak at *m*/*z* 229 due to the additional loss of a catechol unit ([M − H − 198 − 110]^−^). Peak **26** also exhibits an adduct at *m*/*z* 1075 (2[M − H]^−^). In MS2, the base peak *m*/*z* 493 corresponds to the decarboxylation ([M − H − CO_2_]^−^). MS3 fragments at *m*/*z* 359 and 313 could result from caffeic acid moiety. According to previous works, this last fragment allowed the distinction of isomelitric acid A (peak **26**) [69] from salvianolic acid J (peak **12**) [56].

Peaks **15** and **19** exhibited a UV profile characteristic of phenolic acids, in particular ferulic or caffeic acid derivatives, and shared the fragments at *m*/*z* 359 and 223 in their mass spectra. However, in peak **15,** a molecular ion [M − H]^−^ occurs at *m*/*z* 521, and the MS2 peak base at *m*/*z* 359 indicated the loss of a hexosyl moiety ([M − H − 162]^−^). Further, in peak **19** ([M − H]^−^ at *m*/z 359), the MS2 base peak at *m*/*z* 161 and *m*/*z* 197 are derived from the cleavage of the ester bond and the fragment, and *m*/*z* 179 fragment corresponds to caffeic acid unit. Based on spectral behavior similar to those reported in the literature, peaks **15** and **19** were tentatively assigned as rosmarinic acid hexoside [59] and rosmarinic acid [59,60,73].

Peaks **21, 23**, and **24** have the same pseudomolecular ion [M − H]^−^ at *m*/*z* 717, and the fragments at *m*/*z* 519 and 357. These two last fragments resulted from the losses of a danshensu unit ([M − H − 198]^−^) and a part of caffeoyl moiety ([M − H − 162]^−^), respectively. With the exception of peak **24,** peaks **21** and **23** exhibited similar MS fragmentation patterns and UV profiles being tentatively identified as salvianolic acid B isomers [46,51,66]. However, peak **24** exhibited an MS3 peak base at *m*/*z* 321 that corresponds to the loss of two danshensu units ([M − H − 396]^−^). According to Wang et al. (2012) and Don et al. (2020), this difference allows us to tentatively identify peak **24** as salvianolic acid E [46,68].

Peaks **27** and **28** were tentatively assigned as salvianolic acid A isomers due to their similar UV profile and MS fragmentation pattern, namely: pseudomolecular ion [M − H]^−^ at *m*/*z* 493, and MS2 base peak at *m*/*z* 359 (loss of 134 a.m.u.); MS3 peak base occurs at *m*/*z* 161 resulting from the loss of a danshensu unit ([M − H − 359 − 198]^−^) and the fragment at *m*/*z* 179 indicated the presence of caffeic acid moiety [51,70,74].

#### 3.2.2. Flavonoids

##### Flavone Derivatives

Relatively to the flavonoids, peaks **9**, **10**, **20,** and **22** exhibited spectral characteristics of flavones with only one free hydroxyl in the B ring, with absorption bands near 270 (band II) and 330 nm (band I) [75]. Peaks **9** and **10** exhibited similar MS fragmentation patterns and were tentatively identified as apigenin-6,8-di-C-hexoside isomers. The molecular ion [M − H]^−^ occurs at *m*/*z* 593, being the MS2 peak base *m*/*z* 473, and other fragmentation at *m*/*z* 503, being these fragments ([M − H − 120]^−^ and [M − H − 90]^−^, respectively) typical of di-C-glycosyl flavones fragmentation. MS3 peak base at *m*/*z* 353 ([M − H − 120]^−^) and another fragment at *m*/*z* 383 [M − H − 90]^−^) confirm the presence of another glycosyl moiety [52,53,54]. In peak **20**, the molecular ion [M − H]^−^ occurs at *m*/*z* 445 and MS2 and MS3 peak base at *m*/*z* 269, resulting from the loss of a glucuronyl moiety ([M − H − 176]^−^), releasing the genin (tentatively identified as apigenin). Peak **20** was tentatively identified as apigenin-7-*O*-glucuronide (MW 446) [63,64,65]. Peak **22** exhibited a molecular ion [M − H]^−^ at *m*/*z* 607, and MS2 and MS3 fragments at *m*/*z* 299 and 284 being the former resulting from the loss of deoxyhexosyl-hexose unit ([M − H − 308]^−^). Peak **22** was tentatively identified as diosmetin-7-*O*-deoxyhexosylhexoside (MW 608) [67].

##### Flavanones and Flavanonols Derivatives

Peaks **13**, **14**, **18**, **29**, and **30** presented UV spectral characteristics of flavanones or flavanonols due to their UV spectra profile, exhibiting a major band between 270 and 295 nm and a small secondary band in the range of 310–350 nm [76]. Peak **13** showed an absorption maximum of 285 nm and 322 nm. The molecular ion [M − H]^−^ occurs at *m*/*z* 595, an MS2 peak base *m*/*z* 287 resulting from the loss of a deoxyhexosyl-hexoside moiety ([M − H − 308]^−^), characteristic of *O*-glycosyl flavonoids. An MS3 peak base at *m*/*z* 269 was observed. This peak was tentatively identified as eriodictyol-7-*O*-deoxyhesosylhexoside (MW 596) [57]. Peak **14** presented an absorption maximum of 287 nm and a secondary band of 330 nm. The molecular ion [M − H]^−^ occurs at *m*/*z* 303 and MS2 and MS3 peak bases at *m*/*z* 285 resulting from dehydration ([M − H − 18]^−^, and a fragment at *m*/*z* 241 that corresponds to decarboxylation ([M − H − CO_2_]^−^). This peak was tentatively identified as taxifolin (MW 304) [58]. Peak 18 displayed a pseudomolecular ion [M − H]^−^ at *m*/*z* 609 and MS2 peak base at *m*/*z* 301 resulting from the loss of a desoxihexosyl-hexoside unit ([M − H − 308]^−^), indicating the presence of an *O*-glycoside. MS3 peak base occurs at *m*/*z* 301. This peak was tentatively identified as hesperidin (MW 610) [57,62]. Peak **29** exhibited a pseudomolecular ion [M − H]^−^ at *m*/*z* 255 due to its molecular mass (MW 256) and its UV spectrum; peak **29** was tentatively identified as pinocembrin [71]. Peak **30** presented an absorption maximum of 286 and a shoulder at 304 nm. The molecular ion [M − H]^−^ occurs at *m*/*z* 255, and MS2 and MS3 peak bases, also at *m*/*z* 255. Based on these spectral features, peak **30** was tentatively identified as liquiritigenin (MW 256) [64,72].

##### Flavonol Derivatives

Peaks **16** and **17** presented UV spectral characteristics of flavanols due to their UV spectra profile, exhibiting two bands in the range of 250 to 380 nm. Peaks **16** and **17** were tentatively identified as kaempferol-*O*-deoxyhexosyl-hexoside isomers (MW 594). Both peaks exhibited the same MS fragmentation pattern. The molecular ion [M − H]^−^ occurs at *m*/*z* 593 and MS2 peak base at *m*/*z* 285, resulting from the loss of a deoxyhexosyl-hexoside moiety ([M − H − 308]^−^) releasing the genin (kaempferol). MS3 peak base occurs at *m*/*z* 285, which corresponds to the kaempferol [60,61].

### 3.3. T. capitata EO and HRW Possess Radical Scavenging Potential and Reducing Power

Considering the role of reactive oxygen species in the inflammatory response and the progression of inflammation-related diseases [77], we hypothesized that both *T. capitata* extracts could possess anti-radical properties. As shown in Table 3, we report that, in DPPH assay, both the EO and the HRW present radical scavenging properties, being the HRW more effective than the EO (IC_50_ = 18.86 vs. 156.1 µg/mL). However, in the ABTS assay, the essential oil (IC_50_ = 2.98 µg/mL) was found to have higher scavenging activity relative to the HRW (IC_50_ = 14.96 µg/mL). These differences can be due to the mechanism of the reaction and also to the rate of reaction due to the different kinetics of ABTS and DPPH radicals. Further, the stereoselectivity of the radicals, the solubility of the extracts in different systems, and the structure and type (e.g., functional groups) of bioactive compounds contribute to its possible different behavior in quench [78]. Relatively to the ferric reducing power, the HRW was more efficient in accordance with his TE value.

### 3.4. Thymbra capitata EO and HRW Are Non-Toxic towards Macrophages and Fibroblasts

Envisioning a future pharmaceutical application of the EO and HRW, we first aimed to characterize the safety profile of both extracts towards macrophages and fibroblasts.

**Figure 3:**
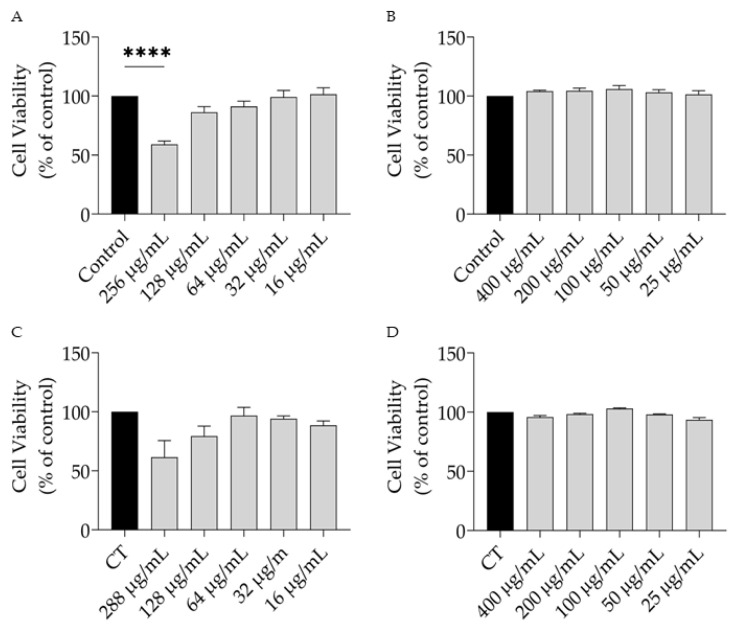
Safety profile of *T. capitata* essential oil (EO) and hydrodistillation residual water (HRW) towards macrophages (**A**,**B**) and fibroblasts (**C**,**D**). **** *p* < 0.0001 when compared to control after one-way ANOVA followed by Dunnet’s multiple comparison test.

As shown in Figure 3, the EO at the highest dose tested (256 µg/mL) present toxicity towards both cell types, whereas the HRW was devoid of toxicity.

Considering these results, the doses of 128 and 400 µg/mL for the EO and HRW, respectively, were selected for further experiments.

### 3.5. Thymbra capitata EO and HRW Exert Anti-Inflammatory Effects in LPS-Stimulated Macrophages

**Figure 4:**
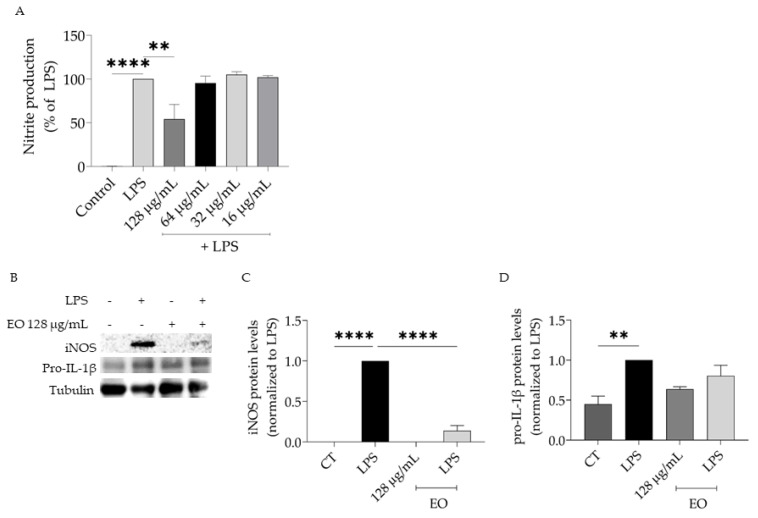
Anti-inflammatory potential of *T. capitata* essential oil (EO). Effect of the EO on nitrite production (**A**) and protein levels of iNOS (**B**,**C**) and pro-IL-1β (**B**,**D**) in lipopolysaccharide (LPS)-stimulated macrophages. ** *p* < 0.01, and **** *p* < 0.0001 when compared to control (CT) or LPS after one-way ANOVA followed by Dunnett’s multiple comparison test.

Due to the relevance of inflammation in evoking inflammaging and considering the anti-inflammatory effects ascribed to this plant, we aimed to assess the effect of *T. capitata* EO and HRW on lipopolysaccharide (LPS)-stimulated macrophages. The presence of LPS for 24 h induced the production of nitric oxide, quantified as nitrites in the culture medium ([NO] = 43.66 ± 4.05 µM). Pre-treating the cells with EO in non-toxic concentrations led to a decrease in quantified nitrites in a dose-dependent fashion (IC_50_ = 103.7 µg/mL), thus suggesting that the essential oil exerts anti-inflammatory effects (Figure 4A). Considering these promising results, we then aimed to highlight the mechanisms of action underlying the reported effect. For that, we analyzed the protein levels of iNOS and pro-IL-1β since these pro-inflammatory proteins are dependent on the NF-κB pathway, which is activated by Toll-like receptors (TLRs), such as TLR4 that is activated by the LPS [79]. As expected, the presence of LPS led to an increase in the protein levels of all tested proteins (Figure 4B–D). The presence of the EO significantly reduced the protein levels of iNOS (Figure 4B,C), thus explaining the reduction in nitrites detected in the culture medium. Regarding IL-1β pro-form, a tendency for the EO to decrease this pro-inflammatory mediator protein levels was observed; however, no significant differences were attained (Figure 4B,D).

**Figure 5:**
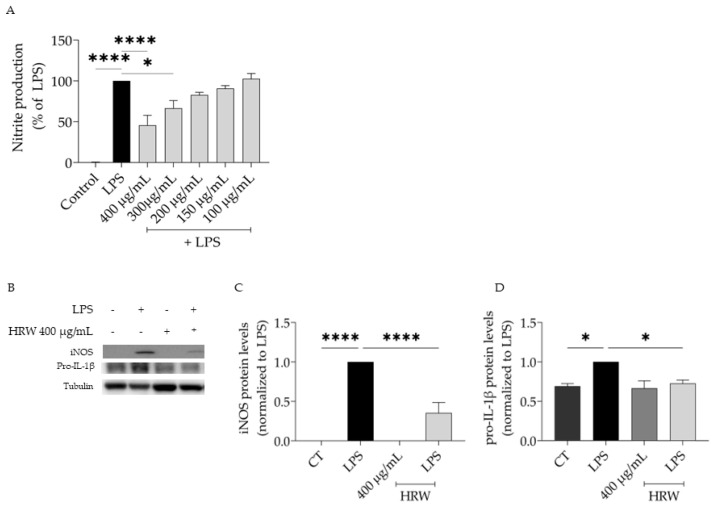
Anti-inflammatory potential of *T. capitata* hydrodistillation residual water (HRW). Effect of the HRW on nitrite production (**A**) and protein levels of iNOS (**B**,**C**) and pro-IL-1β (**B**,**D**) in lipopolysaccharide (LPS)-stimulated macrophages. * *p* < 0.05. Furthermore, **** *p* < 0.0001 when compared to control (CT) or LPS after one-way ANOVA followed by Dunnett’s multiple comparison test.

We further assessed whether the hydrodistillation residual water could exert anti-inflammatory potential. The results achieved demonstrate that this subproduct of essential oil distillation is able to decrease the NO release in LPS-stimulated macrophages (IC_50_ = 377.6 µg/mL, Figure 5A); however, the activity was much lower than reported for the EO. In agreement, the reduction observed in iNOS protein levels was also lower when compared to the EO (Figure 5B,C). Regarding the effect on IL-1β pro-form levels, the HRW exerted a stronger effect than the EO (Figure 5B,D).

### 3.6. Thymbra capitata EO and HRW Differentially Affect Fibroblasts Migration

**Figure 6:**
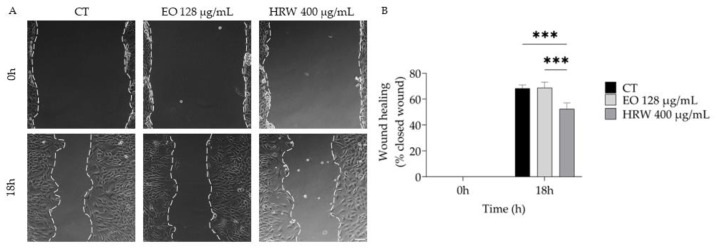
Effect of *T. capitata* EO and HRW on NIH/3T3 fibroblast migration. Representative bright-field images (**A**) and quantification of closed wound using the wound healing size plugin for ImageJ/FIJI (**B**). *** *p* < 0.001 when compared to control (CT) after a two-way ANOVA followed by Sidak’s multiple comparison test.

Due to the traditional uses ascribed for *T. capitata* as a wound healing inductor, we wondered which type of extract could be responsible for this use. As shown in Figure 6, the EO at 128 µg/mL had no impact on cell migration, in contrast with the HRW at 400 µg/mL, which significantly decreased cell migration.

### 3.7. Thymbra capitata EO and HRW Contribute Differentially to Cellular Senescence

**Figure 7:**
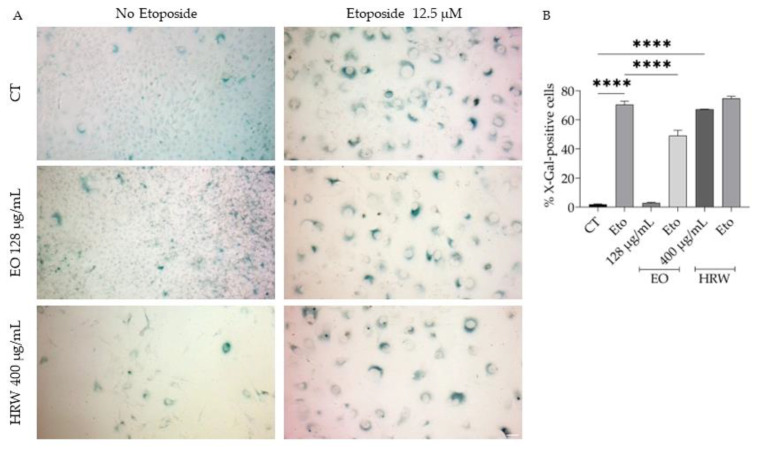
Effect of *T. capitata* EO and HRW on etoposide-induced cellular senescence. Representative bright-field images (**A**) and percentage of X-gal-positive cells after the mentioned treatments (**B**). **** *p* < 0.0001 when compared to control (CT) or etoposide (Eto) after one-way ANOVA followed by Dunnett’s multiple comparison test.

Considering the bi-directional crosstalk between inflammation and cellular senescence [80,81] and that both *T. capitata* extracts exert potent anti-inflammatory effects, we aimed to assess if their presence could decrease cellular senescence, assessed by the evaluation of senescence-associated β-galactosidase activity. As expected, control cells had negligible β-galactosidase activity; however, etoposide (12.5 µM) treatment significantly increased the percentage of β-galactosidase-positive cells significantly increase (Figure 7A,B). Interestingly, when the EO was added to cells in the recovery phase, the β-galactosidase activity was greatly reduced (49% vs. 70%), while the EO alone had no effect on the activity (Figure 7A,B). Unexpectedly, the presence of HRW alone significantly induced the activity of β-galactosidase activity to an extent similar to that of etoposide (67% vs. 70%); however, when cells were pre-treated with etoposide prior to the addition of HRW, the extract did not promote the effect of the positive control (Figure 7A,B).

## 4. Discussion

This study was designed having in mind the anti-inflammatory and wound healing uses ascribed for *T. capitata* and paves the way to further studies validating those uses in pre-clinical and clinical settings. In addition, we report for the first time interesting pharmacological activities to the hydrodistillation residual water (HRW), a by-product from the hydrodistillation procedure that is usually discarded. Furthermore, the chemical composition of the HRW and EO was also highlighted. Overall, the HRW exerts a stronger antioxidant effect in DPPH and FRAP, while the EO was most active in the ABTS scavenging assay. Both extracts reduce relevant pro-inflammatory mediators, specifically NO, iNOS, and pro-IL-1β, probably by blocking NF-kB activation. EO has no effect, whereas HRW reduces cell migration. Anti-senescent properties, assessed by evaluating β-galactosidase activity, were ascribed for EO, while HRW induces cellular senescence.

Regarding the chemical composition of the HRW, this extract is predominantly rich in phenolic acids, particularly rosmarinic acid and salvianolic acids J, B, E, and A. The presence of flavonoids was also reported. The composition of other phenolic extracts from *T. capitata* has already been reported. Indeed, methanolic extracts from *T. capitata* were characterized by rosmarinic acid, salvianolic acid A, salvianolic acid E, hesperidin, eriodictyol, naringenin, and taxifolin [62]. A different study highlighted the composition of acetonic and methanolic extracts of *T. capitata*, with paraben acid, cinnamic acid, and *p*-hydroxybenzoic acid being the main constituents [22]. Other methanolic extracts, also described to be rich in phenolic acids, display gallic, chlorogenic hemihydrate, caffeic, syringic, ferulic, *p*-coumaric, and *trans*-cinnamic acids, as well as flavonoids, particularly myristine, quercetin, kaempferol, catechins, naringenin, and coumarins. Tannic acid and resorcinol were also identified in these extracts [82].

Regarding the essential oil, our results indicated that carvacrol is the main compound, followed by *p*-cymene and γ-terpinene. A very similar composition was reported for samples collected in Portugal [83,84], Malta [85], Spain [19,86,87,88], Morocco [89], and Sicily [90]. These studies highlight that the chemical composition of *T. capitata* growing in the Mediterranean region is highly conserved.

We herein report that *T. capitata* possesses anti-radical properties observed by a strong capacity to scavenge the DPPH and ABTS radical and also a significant reducing power. The HRW showed stronger anti-radical activity in the DPPH assay and also showed a higher reducing power in the FRAP assay, while the EO exhibited stronger anti-radical activity in the ABTS assay. Accordingly, the antioxidant properties of *T. capitata* were previously reported in the literature both for its EO [22,86,91,92,93,94] and non-volatile extracts [18,22,94,95,96]. The anti-radical activity reported for the EO might be attributed to the high amounts of carvacrol. Indeed, carvacrol exerted a stronger antioxidant effect in different assays relative to *p*-cymene, which was devoid of activity [86,97]. Furthermore, this compound has a strong inhibitory effect on the ORAC assay [98], thus validating its potent antioxidant effect. A previous study correlated the phenolic composition of a *T. capitata* extract with its antioxidant effect and reported that rosmarinic acid, caffeic acid, and luteolin-7-methyl-ether positively impact this bioactivity [18], thus suggesting that the activity of the HRW might be attributed to the presence of rosmarinic and caffeic acids. Furthermore, it was reported that rosmarinic acid enhanced the activities of catalase (CAT) and glutathione peroxidase (GSH-Px) while inhibiting the formation of glutathione (GSH) and malondialdehyde (MDA) [99,100,101,102,103]. Considering that the HRW contains both rosmarinic and caffeic acid, it is conceivable that the reported activity might be attributed to their presence; however, the contribution of other compounds cannot be ruled out. Indeed, salvianolic acids have been reported as potent antioxidants [104,105,106,107]. Particularly, salvianolic acid B has been identified as a strong antioxidant agent by inducing the expression of SOD, GSH-Px, and HO-1 while reducing that of NOX-2 and NOX-4 [108]. Salvianolic acid A is also known to exert potent antioxidant effects [109]. The presence of flavonoids also seems to contribute to the reported antioxidant activity of the HRW. The flavone, apigenin, inhibited reactive oxygen species (ROS) production, thus contributing to the reduction of lipid peroxidation and membrane protein damage [110]. It was also reported that apigenin and apigenin 6-C-glucoside-8-C-arabinoside possess antiradical activity and inhibit the XOD enzyme, with the glycosylated derivative being more active [111]. Another glycoside derivative of apigenin, apigenin-7-*O*-glucoside, inhibited ROS production in the same order of magnitude as Trolox. Furthermore, it was stronger than Trolox in protecting erythrocytes from oxidative damage [112]. Kaempferol, kaempferol-7-*O*-rutinoside, kaempferol-7-*O*-rhamnoside, and kaempferol-7-*O*-glucoside exert antiradical effects on both DPPH and ABTS assays, being the aglycone more potent than the glycoside derivatives. Similar effects were reported in LPS-stimulated ROS production [113].

Importantly, our study contributes to highlighting potential mechanisms of action inherent to the anti-inflammatory uses ascribed to *T. capitata*. Indeed, the cooking water and its infusion have been reported to be used as an anti-inflammatory in the Poniente Granadino region in Spain [114]. We report that both the EO and HRW are able to decrease NO release in LPS-stimulated macrophages. Furthermore, we report that both extracts decrease the protein levels of iNOS and pro-IL-1β. Previous studies showed that *T. capitata* EO decreases TNF-α released in LPS-stimulated THP-1 cells [115] and inhibits the activity of lipoxygenase-5 [86,92,94]. Non-volatile extracts are also known to exert anti-inflammatory properties [94,96]. The reported activity can be attributed to the major compounds of both extracts. Indeed, carvacrol has been reported to inhibit lipoxygenase activity [97]. Another study reported that both carvacrol and *p*-cymene inhibit the activity of the same enzyme; however, *p*-cymene had a stronger effect when compared to carvacrol [86]. The anti-inflammatory potential of carvacrol is reported in different models of inflammation, such as IL-1β-stimulated chondrocytes [116], rheumatoid arthritis [117], carcinogenicity associated inflammation in rat colon [118], tonsil epithelial cells [119], paw edema animal model [120], LPS-activated HL-1 cardiomyocytes [121], ovalbumin-induced asthma animal model [122], encephalomyelitis [123] and MNNG-induced gastric carcinogenesis [124]. Rosmarinic acid, the major component in the HRW, is also widely known for its anti-inflammatory properties alone [125,126,127,128,129,130,131,132,133] and in nanovesicles [134] or associated with chitosan [135,136]. The anti-inflammatory property of this phenolic acid is through inhibition of NRLP3 inflammasome [129,134], SIRT1/NF-κB [131], and TLR-4/NF-κB/STAT3 [130]. Inhibition of the NF-κB activation is also reported to be the signaling pathway by which salvianolic acids A and B exert their anti-inflammatory effects [137,138,139,140]. Furthermore, salvianolic acid B also inhibits Mincle/Syk-related pathway [141] and NRPL3 inflammasome [142]. Regarding flavonoids, it was shown that apigenin-7-O-glucoside inhibited NLRP3/caspase-1/NF-κB pathway [112].

Herein, we also report that the EO has no effect on cell migration, while the HRW delays cell migration. In contrast, an ethanolic extract from *T. capitata* is reported to induce wound healing in a wound excision animal model [95]. These opposing effects between the HRW and the ethanolic extract might be attributed to their distinct chemical composition, with the HRW being rich in phenolic compounds, whereas the ethanolic extract was predominantly characterized by lipophilic compounds (tetratria contane, camphor, and terpineol). Concerning carvacrol, the major compound of the EO, its effect on cell migration depends on the cell type. In models of open wounds, the compound promotes wound healing [143,144], while in cancer cells, carvacrol delays cell migration [145,146,147]. Our results suggest that in a complex mixture of compounds, such as an EO, some compounds might antagonize the effect of carvacrol, thus decreasing its pro-migratory properties. Similar effects were reported for rosmarinic acid, promoting cell migration in wound models [148,149] and presenting an inhibitory effect in cancer cells [150,151,152,153,154]. Salvianolic acids A and B are known to induce [155,156,157,158,159] as well as to delay [160,161,162] cell migration. The aglycone apigenin is also reported to promote wound healing in a variety of models [163,164,165,166,167] and to prevent cell migration in cancer cells [168,169,170,171,172,173,174]. Considering these results, it is conceivable that the presence of all these phenolic compounds in the HRW might work antagonistically, thereby decreasing the wound-healing properties of the isolated compounds. Furthermore, and specifically for apigenin, only glycosidic derivatives were found in HRW, which could be devoid of activity due to the presence of the glycosidic moieties.

We also reported here for the first time the anti-aging potential of *T. capitata* EO. The anti-aging potential of the EO might be attributed to the high amounts of carvacrol since it is widely known to exert anti-aging properties. Indeed, carvacrol prevents age-related oxidative damage [175], promotes type I collagen expression [176], inhibits tyrosinase activity [177], thus showing protection against age-related melanogenesis dysregulation, and also inhibits the activity of collagenase, elastase, and hyaluronidase [178] showing skin-aging protection effects. We report that the HRW promotes cellular senescence, suggesting its potential interest in cancer therapies, but further studies should be performed to prove its anti-tumoral effect. Several phenolic compounds present in the HRW possess anti-aging potential. Indeed, rosmarinic acid increases lifespan in *C. elegans* [99] and in an animal model of familial amyotrophic lateral sclerosis [179] and protects cells from UV radiation-induced aging [180,181] as well as in other models of cell senescence [102,182,183,184]. Salvianolic acid B [185,186,187] and apigenin [188,189,190,191,192,193,194] also exert strong anti-aging effects. Considering the results of the isolated compounds, it would be expected that the HRW would prevent cell senescence; however, our results show that the HRW alone induces cell senescence, thus suggesting that these compounds can have antagonistic effects when in a complex mixture. Additionally, the glycosylation of apigenin might also cause the loss of anti-aging potential or even induce cell senescence, and other minor compounds might exert a strong pro-senescent effect, thus counteracting the anti-senescent effect of these compounds. Further studies should be performed to thoroughly evaluate these hypotheses.

## 5. Conclusions

The present study contributes to a better understanding of some of the traditional uses ascribed for *T. capitata*, particularly those related to anti-inflammatory and wound healing uses. Furthermore, we report, for the first time, the anti-senescence potential of the EO, which, combined with the anti-inflammatory activity, supports its anti-aging properties by mitigating two hallmarks of aging and, therefore, with potential interest for the cosmetic industry. In addition, HRW demonstrated inhibitory effects on cell migration, induction of cell senescence, and anti-inflammatory activities, biological activities of interest to be further exploited in the context of anti-cancer therapies.

Overall, the results herein presented highlight the industrial interest of this plant, adding value to a by-product of the hydrodistillation of the EO usually discarded and concomitantly promoting the symbiotic existence between plants and pharmaceutical/cosmetic industries towards the development of a sustainable green bioeconomy with a decreased environmental footprint.

## Figures and Tables

**Table 1:** Chemical composition of the essential oil from *T. capitata*.

Compound *	RI SPB-1 ^a^	RI SW 10 ^b^	Peak Area (%)
α-Thujene	922	1029	0.4
α-Pinene	930	1030	0.3
Oct-1-en-3-ol	956	1440	0.1
Sabinene	964	1128	<0.05
β-Pinene	970	1118	0.3
Myrcene	980	1161	1.1
α-Phellandrene	997	1171	0.1
3-Carene	1003	1155	0.1
α-Terpinene	1010	1187	0.9
*p*-Cymene	1011	1275	4.8
Limonene	1020	1206	0.1
β-Phellandrene	1020	1215	0.1
Z-β-Ocimene	1025	1235	<0.05
E-β-Ocimene	1035	1253	0.1
γ-Terpinene	1046	1249	4.0
*trans*-Sabinene hydrate	1050	1459	0.1
Cymenene	1073	1440	0.1
*cis*-Sabinene hydrate	1080	1544	0.2
Linalool	1081	1543	1.5
*trans*-*p*-2-menthen-1-ol	1122	1623	<0.05
Borneol	1144	1695	0.1
Terpinene-4-ol	1158	1597	1.4
*trans*-Dihydrocarvone	1167	1602	<0.05
α-Terpineol	1169	1692	0.1
Neral	1214	1679	<0.05
Geraniol	1233	1842	0.1
Geranial	1242	1730	0.1
Thymol	1268	2183	0.1
Carvacrol	1275	2212	78.5
*E*-Caryophyllene	1408	1590	2.4
Aromadendrene	1425	1600	<0.05
α-Humulene	1443	1662	0.1
*Allo*-aromadendrene	1445	1636	0.1
Bicyclogermacrene	1481	1726	0.1
Caryophyllene oxide	1557	1968	0.2
Total identified			97.8

* compounds listed in order of elution in the SPB-1 column. ^a^ RI SPB 1: GC retention indices on the SPB-1 column. ^b^ RI SW 10: GC retention indices on the Supelcowax-10 column.

**Table 2:** Compounds identified in hydrodistillation residual water from *T. capitata* by HPLC-PDA-ESI/MSn.

Peak	Partial Identification	R_t_ (min.)	λ_max._ by HPLC/PDA (nm)	[M − H]^−^	MS^2^	MS^3^	Refs.
**1**	Citric acid isomer	1.77	-	191 (100)	[191]: 173 (40),111 (100)	[191 111]: 111 (100)	[44,45]
**2**	Citric acid isomer	2.52	-	191 (100)	[191]: 173 (40), 111 (100)	[191 111]: 111 (100)	[44,45]
**3**	Danshensu	5.62	233, 280 max	197 (100)	[197]: 179 (100)	[197 179]: 135 (100)	[46]
**4**	Salvianolic acid F isomer	9.73	235 max, 281, 310	313 (100)	[313]: 203 (60), 269 (100), 313 (75)	[313 269]: 269 (100)	[47,48]
**5**	Salvianolic acid F isomer	11.39	250 max, 288,310, 335	313 (100)	[313]: 203 (50), 269 (100), 313 (45)	[313 269]: 269 (100)	[47,48]
**6**	Vanillic acid	12.33	234, 284 max, 325	167 (100)	[167]: 123(60), 152 (5), 167(100)	[167 167]: 123 (30), 167 (100)	[49]
**7**	Caffeic acid	15.05	238, 291 sh, 322 max,	179 (100)	[179]: 179 (40), 135 (100)	[179 135]: 135 (100)	[50]
**8**	Prolithospermic acid	18.02	235, 261 max, 299 sh	357 (100)	[357]: 357 (15), 342 (35), 313 (100), 269 (25), 203 (38)	[357 313]: 295 (100), 269 (75), 203 (85)	[51]
**9**	Apigenin-6,8-di-C-hexoside isomer	18.61	235, 271, 333 max,	593 (100)	[593]: 503 (32), 473 (100)	[593 473]: 383 (12), 353 (100)	[52,53,54]
**10**	Apigenin-6,8-di-C-hexoside isomer	19.26	236 max, 253, 280 sh, 337	593 (100)	[593]: 593 (38), 503 (35), 473 (100), 383 (10), 353 (20)	[593 473]: 473 (10), 383 (20), 353 (100)	[52,53]
**11**	Salvianolic acid K	20.46	235, 270 max, 285 sh, 324	555 (100)	[357]: 357 (25), 339 (100), 247 (25),163 (15)	[357 339]: 339 (30), 321 (26), 295 (100), 185 (8)	[55]
**12**	Salvianolic acid J	21.50	285, 342 max	537 (100)	[537]: 493 (9), 339 (100)	[537 339]: 339 (89), 295 (57), 277 (8), 229 (100)	[56]
**13**	Eriodictyol-7-*O*-deoxyhesosylhexoside	21.95	235, 285 max, 299 sh, 322	595 (100)	[595]: 287 (100)	[595 287]: 287 (67), 269 (100), 243 (59)	[57]
**14**	Taxifolin	22.65	235, 287 max, 330 sh	303 (100)	[303]: 285 (100)	[303 285]: 285 (100), 241(85), 175(42)	[58]
**15**	Rosmarinic acid hexoside	24.33	237, 287, 320 max	521 (100)	[521]: 359 (100)	[521 359]: 223 (100)	[59]
**16**	Kaempferol-*O*-deoxyhexosyl-hexoside isomer	25.21	253, 344 max	593 (100)	[593]: 593 (21), 285 (100)	[593 285]: 285 (100)	[60,61]
**17**	Kaempferol-*O*-deoxyhexosyl-hexoside isomer	26.32	253, 344 max	593 (100)	[593]: 593 (35), 285 (100)	[593 285]: 285 (100)	[60,61]
**18**	Hesperidin	27.27	234, 284 max, 325 sh	609 (100)	[609]: 301 (100)	[609 301]: 301 (100), 286 (49), 242 (23)	[57,62]
**19**	Rosmarinic acid	27.88	238 sh, 253, 299, 327 max	359 (100)	[359]: 223 (20), 197 (35), 179 (40), 161 (100)	161 (100)	[59,60]
**20**	Apigenin-7-*O*-glucuronide	29.09	238, 255, 281, 341 max	445 (100)	[445]: 269 (100), 175 (20)	[445 269]: 269 (100)	[63,64,65]
**21**	Salvianolic acid B isomer	29.33	287, 330 max	717 (100)	[717]: 555 (15), 519 (100), 475 (10), 357 (5)	[717 519]: 475 (40), 357 (100)	[51,66]
**22**	Diosmetin-7-*O*-deoxyhexosylhexoside	29.87	251, 267, 338 max	607 (100)	[607]: 299 (100), 284 (20)	[607 299]: 299 (90), 284 (100)	[67]
**23**	Salvianolic acid B isomer	30.35	238, 286, 330 max	717 (100)	[717]: 555 (10), 519 (100), 357 (8)	[717 519]: 357 (100)	[46,51,66]
**24**	Salvianolic acid E	31.19	255, 284, 318 sh	717 (100)	[717]: 519 (100)	[717 519]: 357 (15), 339 (20), 321 (100)	[46,68]
**25**	Unknown	31.66	236 max, 284, 327	1075 (100)	[1075]: 555 (18), 519 (100), 339 (15)	[1075 519]: 339 (100)	-
**26**	Isomelitric acid A	33.18	291, 327 max	1075 (20), 537 (100)	[537]: 493 (100), 359 (15)	[537 493]: 313 (5), 359 (100)	[69]
**27**	Salvianolic acid A isomer	34.21	238, 286 max, 321 sh	493 (100)	[493]: 359 (100)	[493 359]: 223 (25), 197 (28), 179 (45), 161(100)	[51,70]
**28**	Salvianolic acid A isomer	36.35	288, 323 sh	493 (100)	[493]: 359 (100)	[493 359]: 223 (25), 197 (30), 179 (50), 161 (100)	[70]
**29**	Pinocembrin	50.71	289 max, 330 sh	255 (100)	[255]: 255 (100)	[255 255]: 255 (100)	[71]
**30**	Liquiritigenin	52.41	234, 268 max, 304 sh	255 (100)	[255]: 255 (100), 136 (15)	[255 255]: 136 (10), 255 (100)	[64,72]
**31**	Unknown	55.87	276 max, 312 sh	241 (100)	[241]: 241 (100)	[241 241]: 241 (100)	-
**32**	Unknown	58.02	256, 342 max	239 (100)	[239]: 239 (100)	[239 239]: 239 (100)	-

**Table 3:** Antioxidant activity of essential oil (EO) and hydrodistillation residual water (HRW) from *T. capitata* by DPPH, ABTS, and FRAP assays.

Method	Sample	IC_50_ (μg/mL) ^a^	TE (μM/μg Extract) ^b^
**DPPH**	EO	156.1 ± 1.304	1.92 ± 0.04
	HRW	18.86 ± 1.076	3.01 ± 0.04
**ABTS**	EO	2.98 ± 0.20	7.87 ± 0.04
	HRW	14.96 ± 0.45	0.43 ± 0.02
**FRAP**	EO	-	0.32 ± 0.03
	HRW	-	1.85 ± 0.03

^a^ Expressed as mean ± SD of three independent experiments, performed in duplicate. ^b^ Trolox Equivalent.

## Data Availability

Data will be made available upon request.
